# A Self-Paced, Web-Based, Positive Emotion Skills Intervention for Reducing Symptoms of Depression: Protocol for Development and Pilot Testing of MARIGOLD

**DOI:** 10.2196/10494

**Published:** 2018-06-05

**Authors:** Elaine O Cheung, Elizabeth L Addington, Sarah M Bassett, Stephanie A Schuette, Eva W Shiu, Michael A Cohn, Yan Leykin, Laura R Saslow, Judith T Moskowitz

**Affiliations:** ^1^ Department of Medical Social Sciences Osher Center for Integrative Medicine Northwestern University Feinberg School of Medicine Chicago, IL United States; ^2^ Department of Psychology and Neuroscience Duke University Durham, NC United States; ^3^ Osher Center for Integrative Medicine University of California, San Francisco San Francisco, CA United States; ^4^ Palo Alto University Palo Alto, CA United States; ^5^ Department of Health Behavior and Biological Sciences School of Nursing University of Michigan Ann Arbor, MI United States

**Keywords:** emotions, depression, telemedicine, happiness, mobile apps

## Abstract

**Background:**

Living with elevated symptoms of depression can have debilitating consequences for an individual’s psychosocial and physical functioning, quality of life, and health care utilization. A growing body of evidence demonstrates that skills for increasing positive emotion can be helpful to individuals with depression. Although Web-based interventions to reduce negative emotion in individuals with depression are available, these interventions frequently suffer from poor retention and adherence and do not capitalize on the potential benefits of increasing positive emotion.

**Objective:**

The aim of this study was to develop and test a Web-based positive emotion skills intervention tailored for individuals living with elevated depressive symptoms, as well as to develop and test enhancement strategies for increasing retention and adherence to that intervention.

**Methods:**

This study protocol describes the development and testing for Mobile Affect Regulation Intervention with the Goal of Lowering Depression (MARIGOLD), a Web-based positive emotion skills intervention, adapted for individuals with elevated depressive symptomatology. The intervention development is taking place in three phases. In phase 1, we are tailoring an existing positive emotion skills intervention for individuals with elevated symptoms of depression and are pilot testing the tailored version of the intervention in a randomized controlled trial with two control conditions (N=60). In phase 2, we are developing and testing three enhancements aimed at boosting retention and adherence to the Web-based intervention (N=75): facilitator contact, an online discussion board, and virtual badges. In phase 3, we are conducting a multifactorial, nine-arm pilot trial (N=600) to systematically test these enhancement strategies, individually and in combination. The primary outcome is depressive symptom severity. Secondary outcomes include positive and negative emotion, psychological well-being, and coping resources.

**Results:**

The project was funded in August 2014, and data collection was completed in May 2018. Data analysis is currently under way, and the first results are expected to be submitted for publication in 2018.

**Conclusions:**

Findings from this investigation will enable us to develop an optimal package of intervention content and enhancement strategies for individuals with elevated symptoms of depression. If this intervention proves to be effective, it will provide a cost-effective, anonymous, appealing, and flexible approach for reducing symptoms of depression and improving psychological adjustment through increasing positive emotion.

**Trial Registration:**

ClinicalTrials.gov NCT01964820 (Phase 1); https://clinicaltrials.gov/ct2/show/NCT01964820 (Archived by WebCite at http://www.webcitation.org/6zpmKBcyX). ClinicalTrials.gov NCT02861755 (Phase 2); https://clinicaltrials.gov/ct2/show/NCT02861755 (Archived by WebCite at http://www.webcitation.org/6zpmLmy8k).

**Registered Report Identifier:**

RR1-10.2196/10494

## Introduction

### Background

According to the Centers for Disease Control and Prevention, 7.6% of Americans reported moderate to severe symptoms of depression between 2009 and 2012 [1]. Living with elevated symptoms of depression can have debilitating consequences for an individual’s psychosocial and physical functioning, quality of life, and health care utilization [2-4]. Moreover, elevated depressive symptomatology is a significant risk factor for developing major depression [5,6]; has been associated with increased cardiovascular morbidity and mortality [7], risk of disability [8]; and carries an estimated annual economic cost of US $210.5 billion [9]. However, the majority of Americans with elevated symptoms of depression go untreated or undertreated [10]; many individuals lack access to treatment or do not utilize available services [11-13]. Researchers have begun advocating for early intervention in the prevention of depression, highlighting the importance of targeting at-risk subgroups [14,15] such as those with elevated symptoms of depression [5].

Most research has focused on the role of negative emotion in depression, while largely ignoring the role of positive emotion. However, there is considerable evidence suggesting that positive and negative emotion are not simply opposite ends of a single continuum; rather, positive and negative emotion appear to be independent of one another [16,17], can be experienced concurrently [18-20], and positive emotion appears to play a unique role in influencing physical, psychological, and social functioning, over and above the effects of negative emotion [21-23]. In fact, emerging evidence suggests that low positive emotion, in particular, plays a uniquely important role in predicting depressive symptomatology, independent of negative emotion [24-27]. For instance, low positive emotion has been found to prospectively predict the initial onset of a depressive episode [28], and the dampening of positive emotion has been linked with increased symptoms of depression [29-32]. These findings, together with a growing body of evidence highlighting the unique benefits of positive emotion for coping with negative life events more generally [33-35], suggest that increasing positive emotion is a promising pathway to target for reducing symptoms of depression.

Indeed, interventions that target increasing the frequency of positive emotion experienced in daily life appear to be helpful for reducing symptoms of depression: a meta-analysis of 25 single and multicomponent interventions focusing on increasing positive emotional states such as gratitude, happiness, and optimism found that positive emotion skills interventions showed a medium effect size for relief of depressive symptoms, with stronger effects for currently depressed participants relative to nondepressed participants [36]. These interventions show comparable efficacy and long-lasting effects as that of psychotherapy or pharmacotherapy treatments [36,37]. Furthermore, positive emotion skills interventions may help counteract the depression-related motivational deficits that can lead to poor adherence and retention in traditional psychological interventions [36,38].

The internet offers a method for delivering psychological interventions that is time- and resource-efficient and has the benefit of providing treatment to those who may otherwise lack access to available services [39,40]. Moreover, Web-based interventions have the potential to overcome many of the barriers to help-seeking that depressed individuals have reported in the past, including cost, a shortage of trained professionals, concerns about anonymity, convenience, perceived stigma, and ease of accessibility [11,13,41]. For the past two decades, a large number of internet-based interventions for depression have been developed and tested [42], and meta-analyses have indicated that such Web-based interventions can be effective at reducing depressive symptomatology [43-45]. However, many Web-based interventions tend to suffer from poorer adherence and retention [37,46,47], and these issues can be exacerbated in depressed samples, potentially because of the specific psychological features of depression, including pessimism, low motivation, loss of energy, and impaired concentration [45,47-49].

Web-based interventions that are supported by a trained professional (eg, having a trained professional associated with the study guide the participant through the intervention content via email or telephone) have been found in meta-analyses to produce larger effect sizes and better adherence relative to Web-based interventions that are self-guided [43,50]. For instance, one meta-analysis found that the average percentage of fully adherent participants (participants who completed all sessions in the intervention) was 26% for self-guided Web-based interventions versus 72% for supported interventions [50]. However, a disadvantage of supported interventions is that they tend to be more time-intensive, costly, and difficult to disseminate relative to self-guided interventions. This research aims to develop and test low-cost, resource-efficient, and scalable strategies for promoting adherence and retention in Web-based interventions.

**Figure 1:**
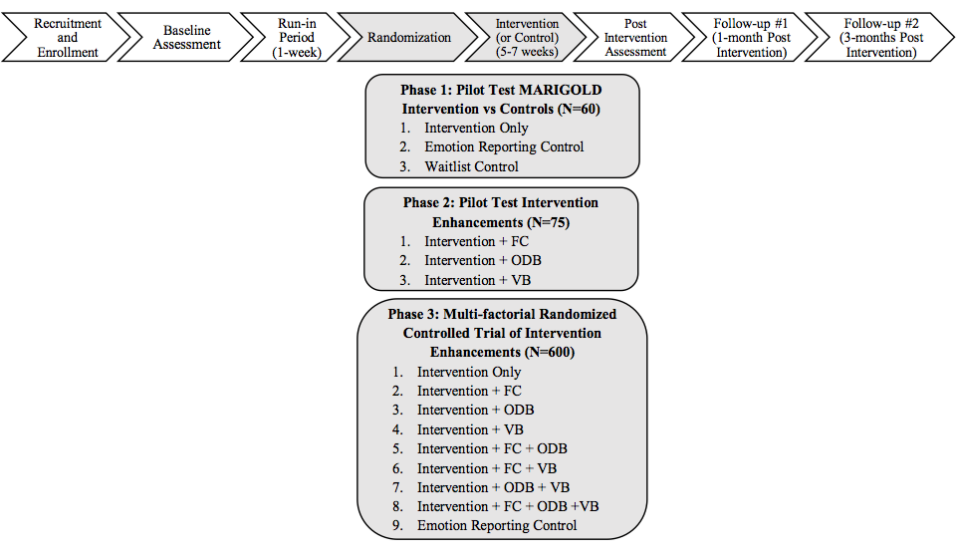
Flowchart of study timeline for phases 1 to 3. FC, facilitator contact; ODB, online discussion board; VB, virtual badges.

Our team has previously developed a multicomponent positive emotion skills intervention for individuals coping with the stress of chronic illness (eg, metastatic breast cancer, HIV, and type 2 diabetes) [51-54]. In our studies, this intervention was found to have reduced depressive symptom severity when administered either in person or online, had high retention even among individuals with high levels of depressive symptoms [51-53], and was associated with increased positive emotion in the midst of stressful life events [52-54]. In this investigation, we are adapting and tailoring the intervention to maximize acceptability and relevance for individuals experiencing elevated levels of depression. In addition, this research aims to address the issues of poor adherence and retention that have plagued previous Web-based interventions.

### Objectives

In this protocol paper, we describe the development and pilot testing of Mobile Affect Regulation Intervention with the Goal of Lowering Depression (MARIGOLD), a Web-based positive emotion skills intervention for individuals with elevated depressive symptomatology. We are adapting an existing multicomponent positive emotion skills intervention [51-54] and tailoring it for individuals experiencing elevated levels of depressive symptoms. In addition, we are developing and testing three enhancements aimed to boost retention and adherence to the internet-based intervention. MARIGOLD is a five-session intervention designed to teach participants eight skills for increasing positive emotion in their daily lives. The intervention development is taking place in three phases (see Figure 1).

In phase 1, we are adapting the Web-based positive emotion skills intervention to maximize the acceptability and relevance of the intervention content for people with elevated symptoms of depression and are pilot testing the tailored version of the intervention. In phase 2, we are pilot testing three enhancements aimed at boosting retention and adherence: (1) facilitator contact, (2) an online discussion board (ODB), and (3) virtual badges. In phase 3, we are conducting a multifactorial, nine-arm pilot trial with 600 participants to systematically test each enhancement strategy, alone and in combination, for retention and adherence. The objectives of this investigation are to (1) test the feasibility and acceptability of a Web-based positive emotion skills intervention tailored for people experiencing elevated depressive symptoms (phases 1-3), (2) test the efficacy of three enhancement strategies (facilitator contact, ODB, and virtual badges) for increasing retention and adherence to the intervention (phases 2 and 3), and (3) test the preliminary efficacy of the positive emotion skills intervention for reducing depressive symptom severity (primary outcome), as well as for improving positive and negative emotion and other indicators of psychological adjustment (eg, perceived stress and meaning and purpose; secondary outcomes). Ultimately, this research seeks to develop an optimized Web-based positive emotion skills intervention, adapted for people experiencing elevated depressive symptoms.

## Methods

### Overview of Study Design

In each of the three phases of the MARIGOLD study, individuals with elevated symptoms of depression participate in the same flow of events (see Figure 1). The three study phases differ primarily in terms of the randomization groups and intervention portions of the study sequence (see Figure 1). Specifically, in phase 1, we are tailoring an existing positive emotion skills intervention for individuals with elevated symptoms of depression and are pilot testing the tailored version of the intervention in a randomized controlled trial (RCT) with two control conditions (N=60). In phase 2, we are developing and testing three enhancements aimed at boosting retention and adherence to the Web-based intervention (N=75): facilitator contact, an ODB, and virtual badges. In phase 3, we are conducting a multifactorial, nine-arm pilot trial (N=600) to systematically test these enhancement strategies, individually and in combination. Each component of the study design is described in further detail below.

### Participants

#### Study Setting

All aspects of the study (recruitment, consent, intervention, and assessments) are conducted online.

#### Eligibility Criteria

To be eligible for participation in any of the three pilot trials (phases 1-3), participants must meet the criteria provided in Textbox 1.

Respondents are ineligible if they have already participated in a prior phase of the study. All procedures are approved by the institutional review boards at participating institutions (University of California, San Francisco, UCSF and Northwestern University), and all participants are providing informed consent. The study was registered with Clinicaltrials.gov; phase 1 at UCSF (#NCT01964820) and phase 2 at Northwestern University (#NCT02861755).

#### Participant Timeline

See Figure 1 for the flow of events for participants in all three phases of the MARIGOLD study.

#### Ethics Approval and Consent to Participate

All procedures are approved by the Institutional Review Boards at participating institutions (UCSF and Northwestern University), and all participants provided informed consent. All staff members underwent updated Human Subjects Research Training either through the Collaborative Institutional Training Initiative or the NIH Human Subjects Training Module.

#### Recruitment and Enrollment

Participants for all three pilot trials (phases 1-3) are recruited online and online consent is obtained from each participant. We use online advertisements on platforms such as Reddit, posting within discussion threads for depression, stress, coping, and psychology. Recruitment links are also posted on Craigslist, Backpage, clinicaltrials.gov, and emailed to potential participants via ResearchMatch. Advertisements contain a link to a Web-based eligibility screener (see inclusion and exclusion criteria above). In phases 1 and 2, eligible individuals are contacted by our research staff via telephone, and the research staff describes the study and answers any questions that participants may have. Following the telephone call, the research staff sends an email to potential participants that includes a link to the online consent form. In phase 3, our team eliminated the telephone call and replaced it with an online instructional video. Potential participants in phase 3 take the Web-based screener, and eligible individuals are automatically directed to a Web page with the instructional video and the Web-based consent form. Phase 3 individuals who are not eligible are automatically notified of their ineligibility, instructed to exit the questionnaire, and are thanked for their time.

#### Run-In Period

Upon consenting to participate in the study, all participants begin the 7-day run-in period to screen participants for compliance. This run-in period must be completed to qualify for randomization. Each day during the 7-day run-in period, participants receive an email with a link to a brief Web-based survey, where they complete a daily emotion report using the revised Differential Emotions Scale (DES; see Multimedia Appendix 1) [35]. Participants who complete at least four emotion reports within the first 7 days are randomized to the study. Participants who do not complete at least four emotion reports within the first week are given a second opportunity to do so. If they do not complete at least four emotion reports within the second 7-day run-in period, they are not randomized in the study. We have used this run-in period in our prior research to screen out noncompliant participants [53].

In addition to the daily emotion reports, participants also complete ecological momentary assessments (EMAs) of their positive and negative emotion [56] during the run-in period. Specifically, participants receive SMS text messages (short message service, SMS) on their mobile devices three times per day for 3 days over the course of the week, prompting them to answer questions regarding their current emotional experience. In phases 2 and 3, participants are also completing measures of their daily negative stressors (assessed using the Daily Inventory of Stressful Events, DISE) [57,58] during the run-in period. The DISE is included in the brief Web-based survey sent to participants (see Multimedia Appendix 1). Although we are collecting EMAs and DISE during the run-in period, we are not using this data to inform whether participants are randomized to the study.

**Eligibility criteria for participation.**

Eligibility criteria

1. Be 18 years or older
2. Have daily access to the internet
3. Own a mobile phone
4. Live in the United States
5. Be fluent in the English language (reading and writing)
6. Have elevated depressive symptoms (8-item Patient Health Questionnaire, PHQ-8, depression score ≥5) [55]

#### Randomization

In phase 1, participants are randomly assigned to one of the three groups using simple randomization. In phases 2 and 3, we are stratifying randomization based on gender and level of depressive symptom severity to ensure sufficient numbers of each group within each condition. We are using the 8-item Patient Health Questionnaire (PHQ-8) [55] as our measure of depressive symptom severity. In phase 2, we are stratifying based on three levels of depressive symptom severity (PHQ-8 score: 5-9, mild; 10-14, moderate; ≥15, severe) [55] and by gender. However, to be more consistent with recommended interpretations of PHQ scores [59], in phase 3, we are stratifying based on four levels of depressive symptom severity (PHQ-8 score: 5-9, mild; 10-14, moderate; 15-19, moderately severe; ≥20, severe) and by gender. The randomization assignments were computer-generated via the study survey software (Qualtrics in phase 1 and Research Electronic Data Capture, REDCap in phases 2 and 3) that accounted for participants’ demographic information and depressive symptom severity scores. The computer-generated randomization was performed by the study coordinator, who was not involved with either the intervention or the assessments, as both were completed online.

#### Assessments and Incentives

For all three pilot trials (phases 1-3), we are administering assessments at the following four time points (see Figure 1): baseline, post intervention (7 weeks post baseline), follow-up 1 (FU1; 1 month post intervention), and follow-up 2 (FU2; 3 months post intervention). The EMA text distribution and data collection are administered using PingQuest [60], a platform for the delivery and management of ecological momentary assessment data, developed by one of the authors (MC). All other assessments are administered online. In phase 1, we are using Qualtrics survey software [61] for Web-based data collection and management. In phases 2 and 3, we are using REDCap [62] for Web-based data collection and management, hosted at Northwestern University. Both Qualtrics and REDCap are secure, Health Insurance Portability and Accountability Act of 1996 compliant, Web-based apps designed to support data collection and management for research studies. Participants for all three pilot trials are compensated up to US $60 total: US $45 for completion of all assessments (US $5 for baseline, US $20 for post intervention, and US $10 for each follow-up) and up to US $15 for completing the first 3 weeks of daily website visits or completing the intervention [35,55,56,63].

#### Positive Emotion Skills Intervention

##### Adapting and Refining Intervention Materials

We have developed a five-session, multicomponent positive emotion skills intervention that can be administered either in person or online. In prior studies, this intervention has shown promise for reducing depressive symptoms and improving psychological adjustment in people coping with the stress of a chronic illness, including women with metastatic breast cancer [51], people newly diagnosed with HIV [54], and people with type 2 diabetes [53]. The intervention involves teaching participants eight empirically based skills to increase the frequency of positive emotion experienced in their daily lives: (1) noticing positive events [64,65], (2) capitalizing on or savoring positive events [66,67], (3) gratitude [68,69], (4) setting and working toward attainable goals [70,71], (5) mindfulness [72,73], (6) positive reappraisal [74,75], (7) focusing on personal strengths [76,77], and (8) small acts of kindness [78,79]. At each session, participants are taught up to three of the skills and are asked to practice each skill as home practice every day until the next weekly session.

In phase 1, we adapted the existing Web-based intervention content to address the specific needs and perspectives of people with depression. First, we modified the intervention content to substitute one of the existing skills in the positive emotion skills intervention, skill # 4: setting and working toward attainable goals, with the skill of behavioral activation. Behavioral activation involves teaching participants techniques to monitor their mood and daily activities and to develop plans to increase the number of activities they engage in through activity scheduling. Behavioral activation shares similar principles as the skill of setting and working toward attainable goals; however, behavioral activation has been studied extensively in the context of depression, and there is a strong evidence base supporting the effectiveness of behavioral activation for reducing symptoms of depression [80-83]. As such, we incorporated techniques and concepts from behavioral activation into the positive emotion skills intervention.

In addition, we adapted the existing Web-based intervention content (eg, text, exercises, and images) to address the specific needs and perspectives of people with depression. Table 1 shows the original intervention content and the new material we added to ensure that our intervention is applicable and useful to individuals experiencing symptoms of depression. The new material is intended to address biases, motivational deficits, or resource limitations (eg, lack of social support) that might make it difficult for participants with elevated depressive symptoms to understand the skills or engage with the exercises.

##### User Testing

Materials were modified and revised in multiple cycles based on feedback from Web-based participants. Specifically, in a prior unpublished study, we collected iterative feedback from 250 Web-based participants with elevated symptoms of depression (PHQ-8 score ≥5) recruited from Amazon’s Mechanical Turk [55]. Consistent with standards of user testing to achieve 95% power to detect text that is offensive, unclear, or otherwise problematic [84], each adapted lesson was shown to at least 20 individuals. Participants read sections of text from the adapted intervention and then completed multiple choice quizzes to assess whether they understood the core idea being presented. Testers also answered Likert-scale questions about whether they found the material enjoyable, understandable, and useful, along with open-ended questions about any material they found offensive or inapplicable. A piece of text was considered acceptable if it met three criteria: (1) at least 80.0% (16/20) of testers passed the comprehension quiz, (2) the average rating for enjoyment and usefulness was above the neutral point on the scale, and (3) no testers found the material seriously offensive or inappropriate. Material that failed these criteria was further revised according to testers’ suggestions for improvement and then resubmitted for testing by at least 10 new participants. For example, early testers viewed several lessons as overly optimistic or difficult; after rewriting these lessons, feedback in subsequent rounds of testing was substantially more positive and met criteria for acceptability.

##### Mobile Affect Regulation Intervention With the Goal of Lowering Depression Intervention Content

The resulting MARIGOLD intervention teaches eight positive emotion skills using lessons and homework that have been tailored to people with elevated symptoms of depression (see Table 1).

**Table 1:** Mobile Affect Regulation Intervention with the Goal of Lowering Depression (MARIGOLD) intervention content adapted for depression.

Session		MARIGOLD^a^ intervention content
**Session 1**		
	Skills 1 and 2: noticing and amplifying positive events; Skill 3: gratitude	Learning to notice small positive moments in life and savoring or amplifying the positive emotional experience. Noticing positive events can help to reduce stress, even in the face of significant life stress Cultivating gratitude as another way to savor positive moments. The potential for gratitude to strengthen our connections with others
	Depression material	Recognizing cognitive biases that can lead to discounting or failing to notice or remember positive events
	Exercises	Daily positive events journal. Daily gratitude journal
**Session 2**		
	Skill 4: activation	Techniques for setting goals that are appropriately challenging but feasible
	Depression material	Ways to practice scheduling activities to break out of a negative spiral Added emphasis on setting small, attainable goals and working up to challenging goals gradually How to select goals that will provide pleasure or mastery experiences
	Exercises	Select a goal for the week and record progress daily
**Session 3**		
	Skill 5: mindfulness	Learning to increase the enjoyment of everyday activities by training ourselves to pay attention, on purpose, in the present moment, with nonjudgment. Learning to practice mindfulness both informally and formally
	Depression material	Using present-focused awareness to combat rumination. Using acceptance to tolerate unpleasant situations with less negative emotion
	Exercises	Select an everyday activity to do mindfully (informal practice). Guided mindfulness meditations (formal practice)
**Session 4**		
	Skill 6: positive reappraisal; Skill 7: strengths	Positive reappraisal as a way to respond to everyday stressors and dispute excessively negative interpretations. Recognizing that it is possible to acknowledge a situation as negative but still appreciate potential benefits (silver linings) or mitigating factors Recognizing personal strengths, skills, or talents
	Depression material	Role of negative cognitions in causing or maintaining depression. Support for acknowledging strengths even in the presence of low self-esteem
	Exercises	Daily reappraisal journal Daily strengths journal (record ways a personal strength or talent was used)
**Session 5**		
	Skill 8: acts of kindness	Engaging in small acts of kindness can help promote happiness and well-being for the individual, in addition to strengthening our relationships
	Depression material	Small, prosocial acts that can be performed even if one is relatively socially isolated
	Exercises	Do something nice for someone else each day and record it in a daily kindness journal

^a^MARIGOLD: Mobile Affect Regulation Intervention with the Goal of Lowering Depression.

The skills are (in the order that they are presented) as follows: (1) noticing positive events, (2) amplifying positive events, (3) gratitude, (4) behavioral activation, (5) mindfulness, (6) positive reappraisal, (7) personal strengths, and (8) acts of kindness. MARIGOLD is delivered as a self-paced, Web-based intervention arranged into five modules containing 1 to 3 skills each. Each module is designed to be completed within 1 week; however, to allow for variations in individual schedules and self-pacing, participants are given a total of 7 weeks to complete the MARIGOLD course. Participants must finish the current week’s skills before the next one’s become available, so the skills are taught in succession. Each skill is associated with a home practice exercise in a journal format, and participants are encouraged to spend approximately 10 min each day reviewing the skill and completing home practice. Participants may also revisit previous weeks’ skills and home practice exercises. After completion of the course, participants maintain access to the course website indefinitely, allowing them to review the skills or continue with their home practice.

In addition, booster sessions that contain brief summaries of the positive emotion skills, along with encouragement and goal setting for continued practice, become available immediately after course completion. For example, if participants are going through a booster session for skill # 8: acts of kindness, they will review a brief summary the skill, as well as a few examples of random acts of kindness. They will also have the chance to review their previous journal entries for the skill and print out a bonus handout on the skill. Finally, in the booster sessions, participants have the opportunity to set a goal to enact the skill. Specifically, participants will be asked to complete the following steps: (1) set a goal for enacting the skill (ie, “I will commit to doing something nice for a friend, loved one, or stranger every day”), (2) a time frame of the commitment (1 week, 2 weeks, 1 month, or 2 months), (3) the frequency of the commitment (more than once a day, once a day, every other day etc), (4) how they plan to keep track of their commitment (write about it on the MARIGOLD website or talk to a friend or activity partner), (5) write down an encouraging note to themselves for when it gets difficult to enact the skill, and (6) digitally sign their name to a summarized page with their commitment. Following these six steps, participants will be emailed a copy of their responses as a reminder.

### Outcomes

#### Retention and Adherence

In this study, we define retention as the number of assessment questionnaires that participants complete. In addition, we define adherence to the intervention in two ways: (1) the number of lesson modules accessed by the participant and (2) the proportion of the intervention content that participants complete (ie, the number of pages that participants view out of the total number of pages in the intervention).

#### Preliminary Efficacy Measures

Assessments include self-report measures of demographic and clinical characteristics, depression, positive and negative emotion, psychological well-being, coping resources, potential moderators, and satisfaction with the intervention. See Multimedia Appendix 1 for the full list of measures. The follow-up interviews in phases 1 and 2 are conducted over the telephone by research staff; if the participant is in the facilitator contact arm (phase 2 only), we ensure that the research staff conducting the follow-up interview is not the same person who was assigned as the participant’s facilitator. In phase 3, the follow-up interviews are delivered as a Web-based survey.

The primary efficacy outcome in the proposed research is depressive symptom severity, which we are measuring using the PHQ-8 [55]. We will also assess the Center for Epidemiological Studies-Depression [63] as an additional measure of depressive symptomatology. Emotion is also a central construct in the proposed research. As such, we are measuring it multiple ways: (1) daily emotion reports [35], completed daily during the run-in period (which serves as the baseline measure) and during the 5- to 7-week intervention period, and also daily, in 1-week bursts at each of the three postintervention assessment periods (post intervention, FU1, and FU2); (2) past-week emotion reports [35] at each of the four assessment periods (baseline, post intervention, FU1, and FU2); and (3) EMA [56] completed three times per day for 3 days per week during the run-in period (which serves as the baseline measure) and for 1-week bursts at each subsequent assessment (post intervention, FU1, FU2).

### Study Design

#### Phase 1: Pilot Test of the Web-Based Positive Emotion Skills Intervention Tailored for Participants With Elevated Symptoms of Depression

Participants (N=60) are randomized into one of three arms: (1) MARIGOLD intervention (N=30), (2) active control (daily emotion reporting during the 5-week intervention period; N=15), or (3) waitlist control (N=15). Participants in the intervention arm receive a five-session positive emotion skills intervention tailored for participants with elevated symptoms of depression (described above). Participants in the daily emotion reporting arm complete the DES [35] daily for the 7-week duration of the intervention period. In past research, we have established that emotion reporting is acceptable as a control condition (retention rates of approximately 80%, similar to the intervention) and that participants perceive it as being beneficial, providing some of the features of a placebo control [53]. Participants in the waitlist control group only complete the assessment questionnaires. Upon completion of the FU1 assessment, participants in both the emotion-reporting and waitlist control arms receive access to the MARIGOLD intervention. Following phase 1, we review the study feedback from telephone interview transcripts and modify and refine the study design, staff training, and intervention content accordingly.

#### Phase 2: Pilot Test of Three Enhancements to Increase Retention and Adherence

In phase 2, we are developing and pilot testing three enhancements that can be added to the Web-based intervention for the purpose of boosting retention and adherence among people with elevated depressive symptoms. Participants (N=75) are randomized into one of three arms, the intervention plus one enhancement: (1) intervention + facilitator contact (N=25), (2) intervention + ODB (N=25), and (3) intervention + virtual badges (N=25). Each enhancement is designed to improve retention and adherence to the intervention by removing practical and motivational barriers to continued engagement. We describe each enhancement strategy below.

##### Facilitator Contact

Even when interventions are entirely computerized, contact with a person associated with the intervention has been found to increase adherence to the study [43,85-89]. Participants assigned to this enhancement arm are contacted once per week by a facilitator, who encourages them to continue with the program and answers any questions they have about the study. Contact is limited to no more than 5 min per week. The facilitator schedules a time each week to call the participant by telephone. If they cannot agree on a time or the participant cannot be reached that week, the facilitator contacts the participant by email. The content of the facilitator script is similar for both telephone and email communication. Before contacting the participant (either via telephone or email), the facilitator checks in on the participant’s progress in the course (eg, the skills accessed that week, home practice completion, and daily emotion survey completion). The facilitator begins the facilitator contact (both telephone and email) by briefly summarizing to the participant their progress in the course that week. Next, facilitators check in with the participant about (1) the skills covered that week, (2) the home practice that week, (3) the daily emotion surveys, and (4) any issues with the technology. Specifically, the facilitator asks participants how each component (eg, skills covered and home practice) went that week, whether the participant experienced any challenges, difficulties, or barriers with each component that week, and whether they had any comments or questions. The facilitator contact ends with the facilitator confirming the time for next week’s facilitator contact call, unless it’s the final week (week 7). When facilitators email participants, facilitators encourage participants to reply with questions, comments, and technology issues. The facilitator does not offer counseling to the participant during the facilitator contact. If the participant begins to request counseling, the facilitator reminds the participant that the facilitator is not in the position to provide advice or therapy to the participant and reinforces that their role is to answer questions, help the participant progress through the course, and discuss challenges, goals, or technology issues. The facilitator encourages the participant to identify opportunities to apply the MARIGOLD skills in their daily life using language from the course content. In cases where the participant is actively seeking counseling, the facilitator recommends that the participant speak with their medical provider, and if they don’t have a medical provider, the facilitator offers resources for the participant to find medical coverage (see training section below).

##### Online Discussion Board

Prior research has found that receiving peer support from other users via a Web-based discussion forum can increase rates of adherence to Web-based interventions [90-94]. Participants assigned to this enhancement arm are able to share questions, experiences, and encouragement with other participants in a pseudonymous Web-based environment (ie, each participant has a consistent username, but it contains no information about their identity). Research assistants serve as moderators, checking the discussion board one or more times weekly to remove posts that are inappropriate (eg, profanity, advertisements, and bullying), to identify any concerns about participant safety or suicidality, to post prompts or suggestions to start discussions, to provide encouragement, and to answer broad questions about the study. Moderators remind users regularly and as needed about guidelines for the discussion board (eg, its supportive purpose and the importance of protecting privacy). They do not provide detailed answers to questions about the intervention or discuss individual exercise responses.

##### Virtual Badges

**Figure 2:**
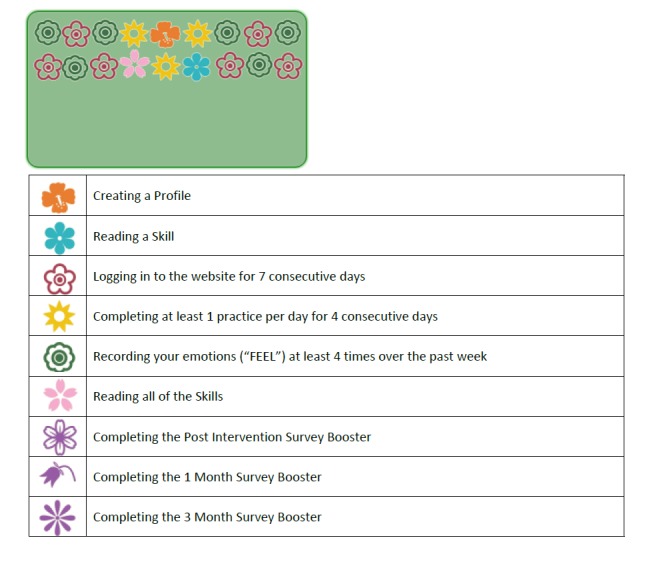
Example garden plot with virtual flower badges and accompanying key in the virtual badges enhancement.

Research shows that learning tasks can be made more engaging and memorable when participants are given proximal goals and benchmarks to strive for and when their accomplishments are reinforced with rewards [95,96]. Participants assigned to this enhancement arm receive virtual flower badges for accomplishing tasks and meeting milestones. These colorful badges are collected on participants’ personal green garden plot, and individuals are encouraged to grow their garden (see Figure 2). Flowers are awarded for different behaviors that can occur once or be repeatable. For example, participants can earn a blue flower each time they read a skill (repeatable) and one pink sunflower after they read all the skills (single occurrence). Completing home practice and logging into the website are also incentivized, with badges awarded for completing at least one home practice exercise for 4 consecutive days (repeatable) and logging into the website for 7 consecutive days (repeatable).

Following phase 2, we review the study feedback from participants and modify and refine the study design, staff training, enhancements, and intervention content accordingly.

#### Phase 3: A Multifactorial Randomized Controlled Pilot Trial to Test Each Intervention Enhancement for Retention and Adherence

Participants (N=600) are randomized to receive the basic intervention, the intervention plus one or more of the three enhancements, or an emotion-reporting only control condition. Specifically, the study is a multifactorial design in which participants are randomized into one of the following nine arms (approximately 67 per arm): (1) intervention only, (2) intervention + facilitator contact, (3) intervention + ODB, (4) intervention + virtual badges, (5) intervention + facilitator contact + ODB, (6) intervention + facilitator contact + virtual badges, (7) intervention + ODB + virtual badges, (7) intervention + facilitator contact + ODB + virtual badges, and (8) emotion reporting only control.

### Training, Fidelity, and Protection of Human Subjects

All staff members receive training on the overall study design and procedures relevant to their staff assignment, including (1) The technology for participant tracking and data collection (eg, REDCap, Qualtrics, and PingQuest); (2) Procedures for serving as a facilitator in the facilitator contact enhancement (eg, training on scheduling telephone and email correspondence with participants, conducting the brief facilitator telephone calls using a standardized facilitator script (see above) to ensure consistent delivery of facilitator contact); (3) Procedures for serving as a moderator of the ODB; (4) Training on the booster sessions; and (5) Protocol for responding to participant suicidality or distress.

Facilitators are trained on how to handle cases of extreme distress or suicidality expressed over the telephone, via email, or on the ODB. In cases of extreme distress or suicidality, the research staff is trained to emphasize to the participant that MARIGOLD is not therapy and to tell participants to contact 911 in the case of an emergency. As part of the distress-suicidality protocol developed by one of the authors (EA), any signs of distress, however small, is reviewed collaboratively by the facilitator, EA, and the study coordinator (ES) to monitor the safety and well-being of participants and to respond appropriately. Facilitators do not offer medical advice and instead, encourage participants to consult their medical provider, offer resources to find a medical provider if he or she does not have one, and provide relevant resources to participants (eg, a suicide hotline).

Training sessions are conducted initially during the study start-up and when onboarding new staff members, on an ongoing basis as the study protocol is updated across the three phases, and on an as-needed basis to address individual cases and procedural issues. All staff members maintained updated Human Subjects Research training either through Collaborative Institutional Training Initiative or the National Institutes of Health (NIH) Human Subjects Research module.

### Planned Analyses

In all three phases of the study, we plan to conduct intention-to-treat analyses to examine (1) Retention and adherence to the intervention and (2) Preliminary efficacy of the intervention. In phase 3, we will have sufficient power to conduct additional analyses examining (3) Moderators of retention and adherence within each randomization arm, (4) Moderators of the primary and secondary efficacy outcomes within each randomization arm, and (5) Whether intervention effects are mediated by increases in positive emotion.

#### Retention and Adherence

Retention will be defined as completing the baseline, postintervention, and follow-up assessments. We will categorize retention at each assessment as a binary outcome and will test for differences in the proportions of participants who are retained at each assessment using a binary logistic regression model using dummy variables to represent each arm. Adherence will be assessed in intervention participants only and measured in two ways: (1) the number of skills accessed and (2) the proportion of the intervention completed (ie, the number of pages viewed out of the total possible pages across all lesson modules in the intervention). In the phase 1 pilot, we will report descriptive statistics of adherence in the intervention arm and will explore whether retention differs as a function of arm (intervention vs emotion-reporting control vs waitlist control) using a binary logistic regression, with dummy variables to represent each arm. In the phase 2 pilot, we will explore whether retention and adherence differ as a function of enhancement type using a binary logistic regression for retention and linear regressions for adherence, with dummy variables to represent each arm. In the phase 3 pilot, we will use additional contrast tests to explore whether retention and adherence differ as a function of enhancement type received (no enhancement, facilitator contact, virtual badges, or ODB), as well as whether receiving certain combinations of enhancements offers additional benefits for increasing retention and adherence.

#### Preliminary Efficacy

Although this investigation is mainly focused on optimizing adherence and retention to the intervention, we will conduct analyses examining the preliminary efficacy of the intervention. Our primary measures of preliminary efficacy will be depressive symptom severity, as assessed by the PHQ-8 [55]. We will also assess change in positive and negative emotion and other indicators of psychological adjustment (eg, perceived stress and meaning and purpose) as secondary outcomes.

For each outcome, we will estimate growth curves within a multilevel modeling (MLM) framework [97] to assess change in each outcome across the four assessment points (baseline, post intervention, FU1, and FU2). MLM offers an approach that accommodates missing data points and nonindependence in observations. We plan to model time at level 1 and randomization arm at level 2 using dummy variables to represent each arm. In phase 1, the primary parameters of interest will be the differences in the magnitude of change in preliminary efficacy outcomes between the intervention arm and each of the control arms over time. In phase 2, the primary parameters of interest will be the magnitude of change in outcomes across all three randomization arms over time. In phase 2, we will also explore whether the three enhancement arms (facilitator contact, ODB, and virtual badges) differ in their magnitude of change in preliminary efficacy outcomes over time. In phase 3, the primary parameter of interest will be the difference in the magnitude of change in preliminary efficacy outcomes over time for the arms that received the intervention relative to the emotion-reporting control. In phase 3, we will also explore whether there are differences in the magnitude of change in outcomes over time as a function of enhancement type (no enhancement, facilitator contact, ODB, or virtual badges), as well as whether receiving certain combinations of enhancements may influence magnitude of change in outcomes over time. We will use significance tests comparing the information criteria of different models to determine which covariates to include in the final model.

#### Moderators of Retention and Adherence

In phase 3, we will assess baseline depressive symptom severity, gender, race or ethnicity, and comfort with technology as moderators of retention and adherence. This will allow us to detect whether certain enhancements to the intervention increase adherence or retention for some subpopulations but not others. For each potential moderator, we will rerun the binary logistic regression analyses with a set of interaction variables between the moderator and the dummy condition variables. Due to power limitations, we will not test all four moderators in the same model or explore any interactions among the moderators.

#### Mediational Analyses

Finally, in phase 3, we will test whether positive emotion mediates any effect of the intervention on overall depressive symptoms. For the mediational analyses, we will combine intervention arms to explore the effects of intervention (regardless of enhancement type). We plan to conduct multilevel moderated mediation analyses [98] using a multilevel structural equation modeling (MSEM) framework [99]. More specifically, mediation effects will be estimated by examining the indirect effect of the intervention on change in depressive symptom severity through the effect of change in the positive emotion. We will test the significance of the specific indirect effect in the MSEM model using the Monte Carlo method with 20,000 bootstraps [100,101]. In addition, we will conduct exploratory multilevel moderated mediational analyses to explore whether improvements in positive emotion mediate the intervention effects on secondary outcomes (eg, psychological well-being, perceived stress, and meaning and purpose).

## Results

The project was funded in August 2014, and data collection was completed in May 2018. Data analysis is currently under way, and the first results are expected to be submitted for publication in 2018.

## Discussion

### Principal Findings

This paper describes the study protocol for the development and pilot testing of MARIGOLD, a Web-based positive emotion skills intervention adapted for individuals with elevated depressive symptomatology. In this work, we are tailoring the intervention content to meet the needs and challenges of individuals with elevated symptoms of depression and are pilot testing the tailored version of the intervention along with three enhancements aimed at boosting retention and adherence to the Web-based intervention content: facilitator contact, ODB, and virtual badges. Ultimately, the goal of this research is to develop an optimized package of relevant content and retention and adherence strategies for individuals with elevated depression.

### Strengths and Limitations

There are a number of strengths to this work. One strength is its innovative focus on positive emotion. Although most psychological interventions for depression tend to target the reduction of negative emotions, this intervention targets increasing positive emotion, which may be an especially promising pathway for helping individuals with elevated depressive symptoms. Another strength of this work is its focus on developing tailored intervention content and enhancement strategies that specifically address the depression-related motivational deficits that can lead to poor adherence and retention. A third strength of this work is systematic development and testing of the intervention over three phases: the development and pilot testing of the intervention content (phase 1), the development and pilot testing of the three enhancement strategies (phase 2), and a multifactorial RCT, systematically testing each enhancement strategy, alone and in combination (phase 3). A fourth strength of this work is the Web-based delivery of the intervention. If the intervention is found to be effective, the self-guided, Web-based delivery of the intervention offers the potential for low-cost, widespread dissemination of the intervention over the internet.

Despite these strengths, potential limitations of this study design should be acknowledged. One significant challenge that faces our project is the potential for our enhancement strategies to actually reduce adherence or efficacy for some users. For example, individuals who are less comfortable or experienced with technology may have difficulty accessing or utilizing the enhancements (eg, their virtual garden plot and ODB). In addition, another limitation is that our follow-up assessments are conducted relatively close to the intervention, with the final follow-up assessment at 3 months post intervention. Future research should include longer follow-up assessments (eg, 1 year post intervention), so that we may examine whether the effects of the intervention persist over an extended period of time.

Furthermore, individuals who have more severe symptoms of depression may be reluctant to engage in Web-based social interactions. We are collecting extensive quantitative and qualitative feedback regarding the intervention content and enhancement strategies, which will give us the opportunity to address any issues that participants may have regarding the accessibility and user-friendliness of the Web-based platform and to remedy any off-putting content or features of the intervention. Additionally, detecting potential moderators of the intervention (eg, comfort with technology and baseline depression severity) will be valuable in itself. Learning more about which intervention features work for which participants will contribute to the development of more sophisticated and targeted interventions in the future.

Finally, research that focuses on the benefits of increasing positive emotion can sometimes be misunderstood as minimizing the significance of depression and its harmful individual and societal consequences. This is not our intent. On the contrary, we understand that depression is real, complex, and painful. We are not encouraging people to simply adopt a *don’t worry-be happy* attitude, nor do we proclaim that increasing positive emotion is a panacea. However, a growing body of evidence demonstrates that increasing positive emotion can promote psychological and physical benefits and catalyze an upward trajectory for people who experience depression or other hardships.

### Conclusions

In sum, the goal of this investigation is to develop and test a Web-based positive emotion skills intervention, tailored for individuals living with elevated depressive symptoms (MARIGOLD). If this intervention proves to be effective, it can provide a cost-effective, anonymous, and flexible approach for reducing depressive symptoms and improving psychological adjustment in individuals living with elevated symptoms of depression.

## Appendix

Multimedia Appendix 1 Study measures and administration frequency.

## Abbreviations

DES: Differential Emotions Scale
DISE: Daily Inventory of Stressful Events
EMA: ecological momentary assessment
FU: follow-up
MARIGOLD: Mobile Affect Regulation Intervention with the Goal of Lowering Depression
MLM: multilevel modeling
MSEM: multilevel structural equation modeling
NIH: National Institutes of Health
ODB: Online discussion board
PHQ-8: 8-item Patient Health Questionnaire
RCT: randomized controlled trial
REDCap: Research Electronic Data Capture
UCSF: University of California, San Francisco
