# Supplementary material for: A Self-Paced, Web-Based, Positive Emotion Skills Intervention for Reducing Symptoms of Depression: Protocol for Development and Pilot Testing of MARIGOLD
Source: JMIR Res Protoc. 2018 Jun 5;7(6):e10494. doi: 10.2196/10494 (PMC6008514; doi:10.2196/10494)
Supplement: Multimedia Appendix 1 [file resprot_v7i6e10494_app1.pdf]

Study measures and administration frequency.

Instrument	Description	Phase			Administered					
		1	2	3	BL	RI	I/C	POST	FU1	FU2
**Demographic and Clinical Characteristics**										
Demographics	Gender, race/ethnicity, age, income	×	×	×	×[a]					
Clinical History	Self-reported clinical information: prior diagnosis of depression, current or past use of medication for depression or anxiety, current or past use of psychotherapy/ counseling	×	×	×	×			×	×	×
**Depression**										
Patient Health Questionnaire (PHQ-8) [56]	8 items measuring depressive symptom severity.	×	×	×	×[a]			×	×	×
Center for Epidemiologic Studies, Depression Scale (CESD) [64]	20 items measuring depressive symptomatology.	×	×	×	×			×	×	×
**Positive and Negative Emotion**										
Past Week Emotion: Modified Differential Emotions Scale (DES) [35]	26 items measuring the frequency of positive and negative emotion experienced over the past week.)	×	×	×	×			×	×	×
Daily Emotion Report: Modified Differential Emotions Scale (DES) [35]	26 items measuring the frequency of positive and negative emotion experienced over the past day.	×	×	×		×	×	×	×	×
Momentary Emotion: Ecological Momentary Assessment of Emotion [57]	5 items measuring the extent to which participants were currently feeling 3 positive and 2 negative emotions (happy, excited, content, anxious, and sad). EMA prompts were delivered via text message 3 times/day for 3 days/week (days 1, 4, and 7).	×	×	×		×		×	×	×

Notes. BL = baseline assessment; RI = run-in period; I/C = intervention (or control) period; POST = post-intervention assessment (approximately 7 weeks post baseline); FU1 = follow-up assessment 1 (1 month post-intervention); FU2 = follow-up assessment 2 (3 months post intervention). [a] Baseline measure administered during the eligibility screening.

Multimedia Appendix (cont'd). *Study Measures*

Instrument	Description	Phase			Administered					
		1	2	3	BL	RI	I/C	POST	FU1	FU2
**Psychological Well-being**										
Perceived Stress Scale (PSS) [103]	10 items assessing participants' stress.	×	×	×	×			×	×	×
Neuro-QOL Positive Affect and Well-being [104]	9 items assessing positive affect, life satisfaction, and an overall sense of purpose and meaning.		×	×	×			×	×	×
PROMIS Meaning and Purpose [105]	8 items assessing meaning and purpose.		×	×	×			×	×	×
Daily Inventory of Stressful Events (DISE) [58,59]	7 items assessing whether the negative daily stressors experienced over the past day.		×	×		×		×	×	×
Psychological Well-being [106]	6 items assessing participants' satisfaction with their life, job, relationships, their general health, sleep quality, and happiness.	×	×	×	×			×	×	×
Pain [107]	Single item assessing the pain experienced during the past week.			×	×			×	×	×
**Coping Resources**										
Positive Skills Usage [52]	10 items assessing the frequency of using the positive emotion skills (e.g., positive reappraisal, gratitude) over the past week.	×	×	×	×			×	×	×
Five Facet Mindfulness Questionnaire (FFMQ) [108]	7 items excerpted from FFMQ, assessing elements of mindfulness (i.e., observing, describing, acting with awareness, non-judging, non-reactivity).	×	×	×	×			×	×	×
Prioritizing Positivity Scale [109]	6 items measuring the extent to which a person seeks out positive emotional experiences when organizing their day-to-day life.		×	×	×			×	×	×
Brief Resilience Scale (BRS) [110]	6 items measuring self-reported resilience.		×	×	×			×	×	×
Depression Self Stigma Scale (DSSS) [111]	32 items measuring self-stigma related to depression.		×		×			×	×	×
Self Stigma Depression Scale (SSDS) [112]	16 items assessing levels of measures shame, self-blame, help-seeking inhibition and social inadequacy regarding depressive symptoms.			×	×			×	×	×

Multimedia Appendix (cont’d). *Study Measures*

Instrument	Description	Phase			Administered					
		1	2	3	BL	RI	I/C	POST	FU1	FU2
**Coping Resources (cont’d)**										
Self-Compassion Scale, Short Form (SCS-SF) [113]	12 items assessing levels of self-compassion.			×	×			×	×	×
Happiness Inducing Behavior Scale (HIBS) [114]	38 items measuring how often participants have completed happiness-inducing behaviors.			×	×			×	×	×
Stress Management Self Efficacy Scale (SMSE) [115]	4 items measuring the perceived ability to cope with stress.			×	×			×	×	×
**Moderators**										
Behavioral Activation for Depression Scale - Short Form [116]	9 items measuring behavioral activation (e.g., accomplishing goals, participating in activities).			×	×					
Conscientiousness [117]	2 items assessing participants’ conscientiousness	×	×	×	×			×	×	×
Comfort with Technology	6 items assessing a participant’s overall frequency of and confidence with technology use			×	×					
Technology Use for the Study	1-item administered assessing the most frequent mode of technology used for the study.							×		
Motivation to Practice Positive Emotion Skills [118]	16 items assessing participants’ motivations (e.g., intrinsic, extrinsic) for using the skills from the MARIGOLD study (intervention only).			×				×	×	×
Expectancy Credibility Scale [119]	6 items assessing participants’ expectancy and credibility beliefs regarding the intervention.			×	×			×	×	×
General Behavior Inventory (GBI) - Mania subscale [120]	7 items assessing symptoms of mania.		×	×	×			×	×	×
**Acceptability**										
Feedback Survey	Quantitative and qualitative items assessing participants’ views of the content they received during the intervention (or control) period and the enhancements (e.g., satisfaction or dissatisfaction with the intervention, and intention to continue using the skills.)	×	×	×				×		

Notes. BL = baseline assessment; RI = run-in period; I/C = intervention (or control) period; POST = post-intervention assessment (7 weeks post baseline); FU1 = follow-up assessment 1 (1 month post-intervention); FU2 =

follow-up assessment 2 (3 months post intervention).[a] The waitlist control group in Phase 1 received the feedback survey after the FU2 assessment. The feedback survey was administered at POST for all other groups in Phases 1-3.

References

56. Kroenke, K., et al., *The PHQ-8 as a measure of current depression in the general population.* Journal of affective disorders, 2009. **114**(1): p. 163-173.

64. Radloff, L.S., *The CES-D scale a self-report depression scale for research in the general population.* Applied psychological measurement, 1977. **1**(3): p. 385-401.

35. Fredrickson, B.L., et al., *What good are positive emotions in crises? A prospective study of resilience and emotions following the terrorist attacks on the United States on September 11th, 2001.* Journal of Personality and Social Psychlogy, 2003. **84**: p. 365-376.

57. Steptoe, A. and J. Wardle, *Positive affect measured using ecological momentary assessment and survival in older men and women.* Proceedings of the National Academy of Sciences of the United States of America, 2011. **108**(45): p. 18244-8.

103. Cohen, S., T. Kamarck, and R. Mermelstein, *A global measure of perceived stress.* Journal of Health and Social Behavior, 1983. **24**: p. 385-396.

104. Salsman, J.M., et al., *Development and validation of the positive affect and well-being scale for the neurology quality of life (Neuro-QOL) measurement system.* Quality of Life Research, 2013. **22**(9): p. 2569-2580.

105. Cella, D., et al., *The Patient-Reported Outcomes Measurement Information System (PROMIS) developed and tested its first wave of adult self-reported health outcome item banks: 2005–2008.* Journal of clinical epidemiology, 2010. **63**(11): p. 1179-1194.

58. Almeida, D.M., E. Wethington, and R.C. Kessler, *The daily inventory of stressful events: An interview-based approach for measuring daily stressors.* Assessment, 2002. **9**(1): p. 41-55.

59. Almeida, D.M., R.S. Stawski, and K.E. Cichy, *Combining checklist and interview approaches for assessing daily stressors: The Daily Inventory of Stressful Events.* The handbook of stress science: Biology, psychology, and health, 2011: p. 583-595.

106. Association, W.V.S., *World values survey.* Wave, 2005. **5**: p. 2005-2008.

107. Butt, Z., et al., *Use of a single-item screening tool to detect clinically significant fatigue, pain, distress, and anorexia in ambulatory cancer practice.* Journal of pain and symptom management, 2008. **35**(1): p. 20-30.

52. Cheung, E.O., et al., *A randomized pilot trial of a positive affect skill intervention (lessons in linking affect and coping) for women with metastatic breast cancer.* Psycho-Oncology, 2016: p. n/a-n/a.

108. Baer, R.A., et al., *Using self-report assessment methods to explore facets of mindfulness.* Assessment, 2006. **13**: p. 27-45.

109. Catalino, L.I., S.B. Algoe, and B.L. Fredrickson, *Prioritizing positivity: An effective approach to pursuing happiness?* Emotion, 2014. **14**(6): p. 1155.

110. Smith, B.W., et al., *The brief resilience scale: assessing the ability to bounce back.* International journal of behavioral medicine, 2008. **15**(3): p. 194-200.

111. Kanter, J.W., L.C. Rusch, and M.J. Brondino, *Depression self-stigma: a new measure and preliminary findings.* The Journal of nervous and mental disease, 2008. **196**(9): p. 663-670.

112. Barney, L.J., et al., *The Self-Stigma of Depression Scale (SSDS): development and psychometric evaluation of a new instrument.* International Journal of Methods in Psychiatric Research, 2010. **19**(4): p. 243-254.

113. Raes, F., et al., *Construction and factorial validation of a short form of the self-compassion scale.* Clinical psychology & psychotherapy, 2011. **18**(3): p. 250-255.
114. Warner, R.M. and K.G. Vroman, *Happiness inducing behaviors in everyday life: An empirical assessment of "the how of happiness".* Journal of Happiness Studies, 2011. **12**(6): p. 1063-1082.
115. Sawatzky, R.G., et al., *Stress and depression in students: the mediating role of stress management self-efficacy.* Nursing research, 2012. **61**(1): p. 13-21.
116. Manos, R.C., J.W. Kanter, and W. Luo, *The behavioral activation for depression scale–short form: development and validation.* Behavior Therapy, 2011. **42**(4): p. 726-739.
117. Gosling, S.D., P.J. Rentfrow, and W.B. Swann, *A very brief measure of the Big-Five personality domains.* Journal of Research in personality, 2003. **37**(6): p. 504-528.
118. Guay, F., R.J. Vallerand, and C. Blanchard, *On the assessment of situational intrinsic and extrinsic motivation: The Situational Motivation Scale (SIMS).* Motivation and emotion, 2000. **24**(3): p. 175-213.
119. Devilly, G.J. and T.D. Borkovec, *Psychometric properties of the credibility/expectancy questionnaire.* Journal of behavior therapy and experimental psychiatry, 2000. **31**(2): p. 73-86.
120. Depue, R.A., et al., *General Behavior Inventory identification of unipolar and bipolar affective conditions in a non-clinical university population.* Journal of Abnormal Psychology, 1989. **98**: p. 117-126.
